# A Computational Study of the Effects of Tachycardia-Induced Remodeling on Calcium Wave Propagation in Rabbit Atrial Myocytes

**DOI:** 10.3389/fphys.2021.651428

**Published:** 2021-04-09

**Authors:** Márcia R. Vagos, Hermenegild Arevalo, Jordi Heijman, Ulrich Schotten, Joakim Sundnes

**Affiliations:** ^1^Simula Research Laboratory, Computational Physiology Department, Lysaker, Norway; ^2^Faculty of Health, Medicine and Life Sciences, School for Cardiovascular Diseases, Maastricht, Netherlands; ^3^Department of Informatics, University of Oslo, Oslo, Norway

**Keywords:** rabbit atrial cardiomyocyte, computational model, spatial calcium dynamics, calcium waves, population of models, tachypacing

## Abstract

In atrial cardiomyocytes without a well-developed T-tubule system, calcium diffuses from the periphery toward the center creating a centripetal wave pattern. During atrial fibrillation, rapid activation of atrial myocytes induces complex remodeling in diffusion properties that result in failure of calcium to propagate in a fully regenerative manner toward the center; a phenomenon termed “calcium silencing.” This has been observed in rabbit atrial myocytes after exposure to prolonged rapid pacing. Although experimental studies have pointed to possible mechanisms underlying calcium silencing, their individual effects and relative importance remain largely unknown. In this study we used computational modeling of the rabbit atrial cardiomyocyte to query the individual and combined effects of the proposed mechanisms leading to calcium silencing and abnormal calcium wave propagation. We employed a population of models obtained from a newly developed model of the rabbit atrial myocyte with spatial representation of intracellular calcium handling. We selected parameters in the model that represent experimentally observed cellular remodeling which have been implicated in calcium silencing, and scaled their values in the population to match experimental observations. In particular, we changed the maximum conductances of ICaL, INCX, and INaK, RyR open probability, RyR density, Serca2a density, and calcium buffering strength. We incorporated remodeling in a population of 16 models by independently varying parameters that reproduce experimentally observed cellular remodeling, and quantified the resulting alterations in calcium dynamics and wave propagation patterns. The results show a strong effect of ICaL in driving calcium silencing, with INCX, INaK, and RyR density also resulting in calcium silencing in some models. Calcium alternans was observed in some models where INCX and Serca2a density had been changed. Simultaneously incorporating changes in all remodeled parameters resulted in calcium silencing in all models, indicating the predominant role of decreasing ICaL in the population phenotype.

## 1. Introduction

Models of cardiac electrophysiology are important tools for studying the mechanisms of heart disease and impaired cardiac cell function, and have been particularly relevant in the study of arrhythmic events such as atrial fibrillation (AF) (Trayanova, 2014; Grandi and Maleckar, 2016; Vagos et al., 2018). An important application of the models is interpretation and translation of knowledge gained from animal experiments performed on a variety of different species. Atrial cardiomyocytes have a less developed T-tubule system than their ventricular counterpart, and in many species this results in markedly different intracellular calcium dynamics. In both cell types calcium enters via L-type calcium channels, locally accumulates close to the membrane, and triggers calcium-induced calcium release from calcium release units (CRU) in the junctional sarcoplasmic reticulum (SR) into the cytosol. The complex T-tubular system of ventricular cells effectively ensures that every CRU is in close proximity with the membrane, and ensures that this process occurs simultaneously throughout the cell. In atrial cells without well-developed T-tubules, only a small fraction of the CRUs are close to the membrane and therefore activated by the initial L-type channel influx. Activation of the remaining CRUs occurs as a calcium wave which propagates from the membrane toward the center of the cell via a regenerative “fire-diffuse-fire” response. As one CRU is activated, calcium diffuses through the cytosol to cause a local concentration rise at the neighboring CRUs, which then reaches the threshold level for activation and releases more calcium and the process repeats resulting in a centripetally propagating calcium wave.

Given the heterogeneous nature of intracellular calcium signaling in atrial myocytes (Trafford et al., 2013), they are prone to developing calcium instabilities, such as local fluctuations in cytosolic calcium levels, which can translate into arrhythmogenic activity (Greiser et al., 2014; Voigt et al., 2014). Furthermore, sustained arrhythmic conditions such as AF can lead to severe electrophysiological remodeling of the atrial myocytes, including up- or down-regulation of numerous ion channels and other membrane transporters. These changes may affect the calcium cycling and propagation within the cell, and may potentially exacerbate repolarization instabilities. For instance, calcium alternans have been extensively observed and implicated as precursors of AF episodes in patients (Narayan et al., 2002).

It has also been shown in rat atrial myocytes that under basal conditions, calcium does not fully propagate toward the center of the cell, mainly due to extensive calcium buffering, and the diffusion barrier of the mitochondria and Serca2a (Bootman et al., 2011). It is believed that this lack of a calcium signal at the inner regions of the cell under normal conditions acts as a reserve to allow calcium release to be increased under conditions of higher contraction demand, such as during β-adrenergic stimulation.

Although atrial myocytes of rabbit and rat species share similar morphological traits, including size and the lack of a well-developed T-tubule system, it has been observed in line scans of rabbit atrial myocytes that calcium propagates in a fully regenerative manner (Greiser et al., 2014), unlike in rat atrial cells. However, the same study showed that replicating chronic AF conditions via rapid pacing of rabbit atrial myocytes resulted in a complete absence of calcium propagation inside the cell, a conditioned termed “calcium silencing.” This behavior was suggested to be a cardio-protective mechanism of the cell to suppress arrhythmogenic after-depolarizations, and has been observed both in rabbit and in AF patient myocytes (Greiser et al., 2014). Additionally, several studies have pointed out the role of calcium buffering in regulating available free calcium in the cell, and ultimately being a promoter of calcium handling abnormalities (Greiser et al., 2014; Smith and Eisner, 2019). However, the exact mechanisms leading to calcium silencing, and their relative importance, remain unclear.

In the present paper we elucidate the underlying mechanisms of calcium silencing by comparing computational model results with experimental observations. We have previously developed a model of the rabbit atrial myocyte with spatial calcium description (Vagos et al., 2020), which was parameterized to match experimentally observed rabbit-specific electrophysiology using a population of models approach. The parameterization resulted in 16 different models of the rabbit atrial cell, each with a unique combination of ion channel maximum conductances, but all closely matching experimentally observed electrophysiological characteristics. All models showed a rabbit-like action potential (AP) and calcium transient (CaT), as well as fully propagating calcium waves. The purpose of the present study is to assess the role of different cellular parameters that are believed to change as a result of rapid atrial pacing (RAP) induced remodeling. By changing their values in the model in one-at-a-time fashion, and applying these changes to all of the 16 models obtained from our previous study (Vagos et al., 2020). We changed the maximum conductances of ICaL, INCX, and INaK, RyR open probability parameters, RyR density, Serca2a density, as well as calcium buffering strength. We chose these specific parameters since all of them were shown to be altered in rabbit atrial myocytes as a consequence of RAP remodeling (Greiser et al., 2014). Furthermore, we scaled these parameters in the model according to experimental data reported in Greiser et al. (2014). Therefore, the changes introduced in the model to incorporate remodeling effects are based on direct measurements rather than indirect estimates from AP or CaT observations.

Furthermore, it has been extensively shown that cardiomyocytes have a large degree of variability in ion channel expression levels and electrophysiological properties. Therefore, the advantage of changing the parameters in 16 different models is that it allows to incorporate electrophysiological variability in the simulations, providing a more comprehensive assessment of the effects of each individual parameter. Using a population of models rather than a single “representative” model, we obtain a more robust and insightful analysis of the model behavior and associated uncertainty. By testing the effects of parameter changes in different model instances it is possible to obtain a more generalized response of the model and to identify possible drivers of the observed responses.

## 2. Methods

### 2.1. Control Model Population

**Figure 1:**
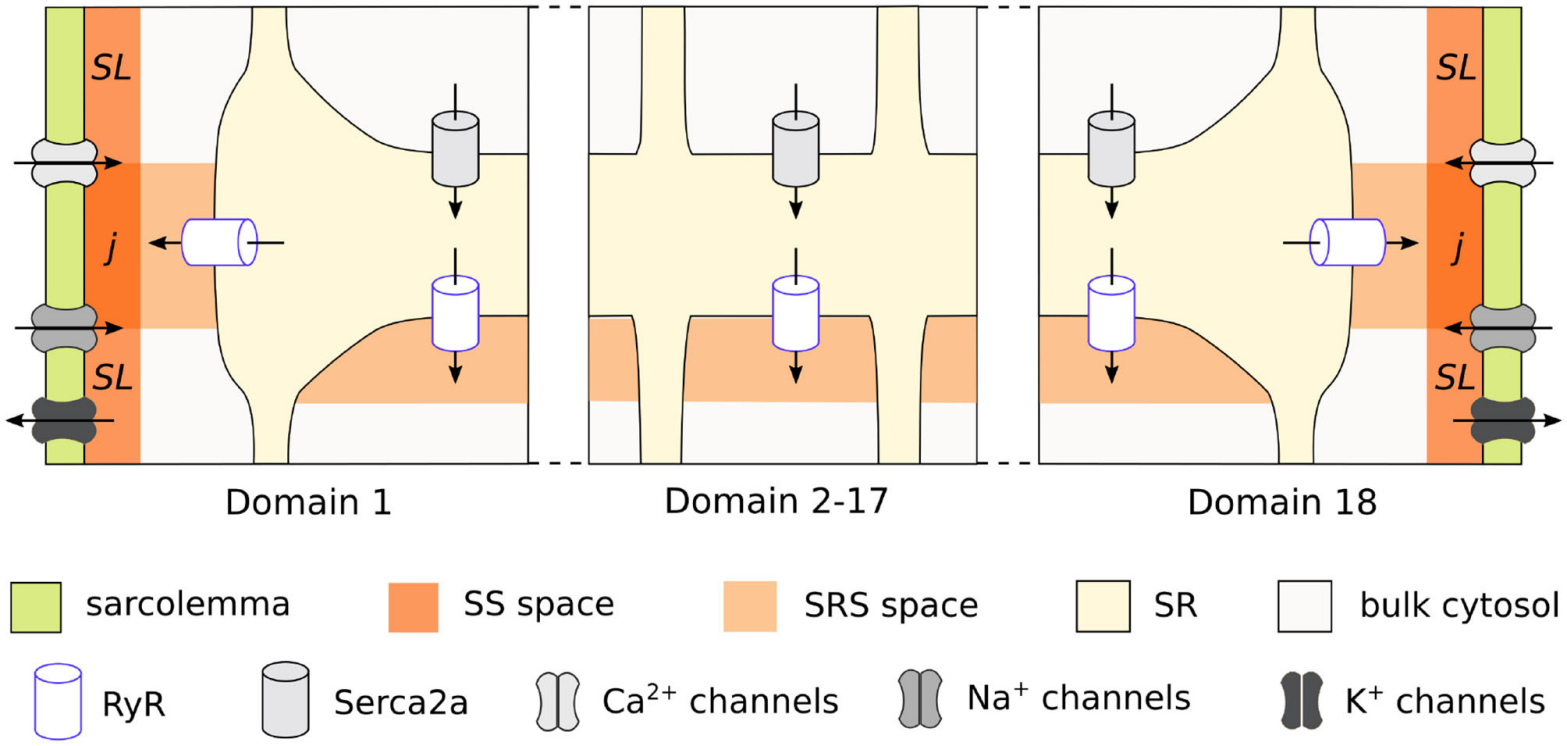
Schematic representation of the rabbit cardiomyocyte model, showing how a transversal cell segment is divided into individual domains. Calcium enters the cell via L-type channels in the membrane domains (1 and 18) and diffuses toward the cell center. Each domain has its own sarcoplasmic reticulum and calcium release units, while the membrane domains also include membrane currents and sub-sarcolemmal spaces.

We used the previously published model of the rabbit atrial cardiomyocyte with spatial description of the calcium handling system, which allows for the simulation of intracellular calcium wave propagation. A complete description of the model development and structure is provided in Vagos et al. (2020). In brief, the model was based on the rabbit atrial myocyte model by Aslanidi et al. (2009), and the human atrial myocyte model by Voigt et al. (2014). It is composed of discrete units, each containing ionic concentrations and cellular structures specific of its location in the cell. Conceptually, the model is based on segmenting the cardiomyocyte into transversal slices, which are further sub-divided into domains in the transversal direction, as illustrated in Figure 1. As described in detail in Vagos et al. (2020), the model distinguishes between the domains close to the membrane and the interior domains. The inner domains contain the cytosol, sarcoplasmic reticulum (SR), a sub-SR space (SRS), and calcium release units (CRU), while the membrane domains also include the sub-sarcolemmal (SL) space, junctional space (j), and associated sarcolemmal currents. The membrane currents include calcium currents ICaL and I_CaT_; the fast sodium current I_Na_; repolarizing K^+^ currents I_to1_, I_Kr_, I_Ks_, and I_K1_, three background currents I_Cab_, I_Nab_, and I_Clb_, as well as the Na^+^-Ca^2+^ exchanger INCX, the Na^+^-K^+^ pump INaK, and the plasmalemmal Ca^2+^ pump I_CaP_. The formulation of the INCX current was adopted from Voigt et al. (2014), while all other ionic current formulations were taken from Aslanidi et al. (2009), and reparameterized in Vagos et al. (2020). Flux of Ca^2+^ between neighboring domains is described by a linear diffusion model adopted from Voigt et al. (2014). The model equations are specified in detail in Vagos et al. (2020), and the source code is available^1^.

**Figure 2:**
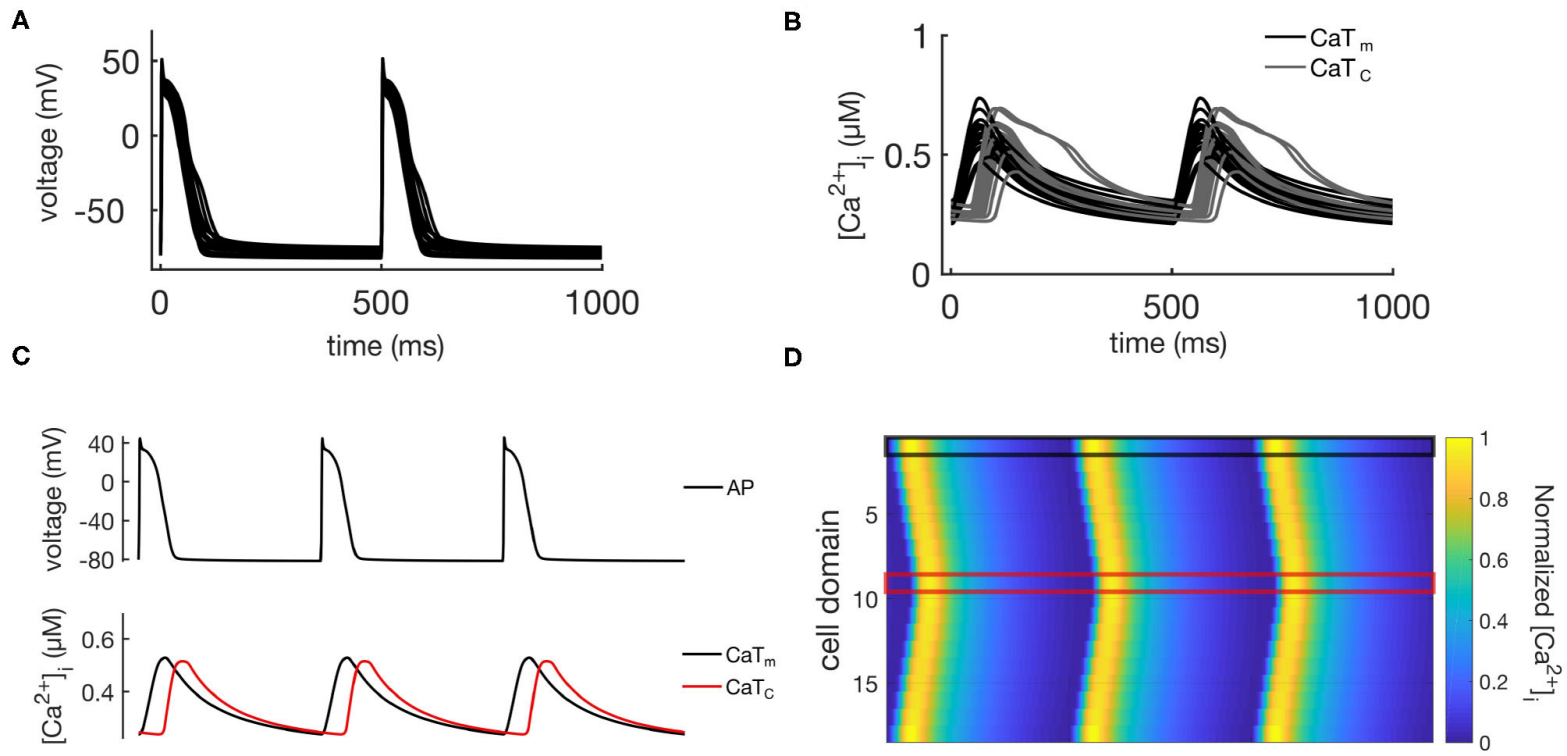
Illustration of the behavior of the control population. **(A)** Shows APs for all 16 models, while the corresponding CaTs from the central and membrane domains are shown in **(B)**. **(C)** Shows the same data for a single representative model, while **(D)** shows spatio-temporal plots of the calcium propagation for the same model.

The calibrated population of models developed in Vagos et al. (2020) consisted of 16 models with rabbit-like electrophysiological parameters: (1) fully regenerative calcium wave propagation from periphery to center of the cell; (2) absence of alternans or afterdepolarizations; and (3) AP and whole cell CaT morphologies within the physiological ranges experimentally observed in rabbit atrial myocytes. The models differed only in the values of maximum conductances of the ionic currents, thus capturing the natural variability observed in atrial cells. In the following we will refer to these 16 models as the “control population,” and its main characteristics are illustrated in Figure 2. Figure 2A shows APs for all 16 models, Figure 2B shows the CaTs from the membrane domains (CaTm) and the innermost domains (CaTc), while Figures 2C,D show an exemplary AP, CaTs, and calcium wave from one of the models. We refer to Vagos et al. (2020) for a complete specification of the control model population, including parameter values for all 16 models.

### 2.2. Incorporating RAP Remodeling

An experimental study has shown that rabbit atrial myocytes subjected to rapid atrial pacing (RAP), at 10 Hz for 5 days, developed an array of cellular alterations at the structural and biochemical levels (Greiser et al., 2014). Most notably, observations included decreased (1) ICaL and (2) INaK currents, (3) increased INCX, (4) reduced Serca2a density, (5) increased ryanodine receptor (RyR) open probability, (6) decreased RyR cluster density, and (7) increased calcium buffering strength. The membrane currents and Serca2a density were modified by g the maximum fluxes, to increase the RyR open probability we scaled parameters RyR_P[0], RyR_P[1], and RyR_P[5], as suggested in Voigt et al. (2014), decreased RyR density was incorporated by scaling RyR_P[11] and the NRyRs parameter, while buffering was increased by a scaling of the parameter Buff_factor. The parameter changes are specified in Table 1, and we refer to Vagos et al. (2020) and Voigt et al. (2014) for a specification of the model equations and parameters. These cellular alterations have been proposed as being a cardioprotective mechanism against arrhythmogenic calcium activity (Greiser, 2017). We introduced these alterations in the control model population defined above to assess the effects of each alteration on the action potential (AP) and calcium wave propagation dynamics, and also the effect of all the changes combined. The remodeling was incorporated by scaling the baseline values of the model parameters that modulate the affected cellular mechanisms to match experimental measurements. Each of the seven alterations listed above were implemented individually to the 16 models of the control population, and we also created one population that incorporated all the listed modifications collectively, resulting in eight remodeled populations with 16 models in each. The parameter changes applied for each of the remodeled populations are specified in Table 1. By applying the remodeling to a calibrated control population rather than a single representative model, we were able to capture more of the natural variability and parameter uncertainty and provide a more complete picture of the potential effect on calcium dynamics.

**Table 1:** List of the 8 different RAP-induced remodeled populations, and the parameters changed in the rabbit atrial myocyte model to represent remodeling in each case.

**Remodeled population**		**Parameters**	**Parameters definition**	**Baseline value**	**Scaling**
1	Down-regulation of ICaL	GCaL	Maximum conductance of the T-type calcium channel	see Vagos et al. (2020)	0.4
2	Up-regulation of INCX	I*max*NCX	Maximum flux of the Ca^2+^-Na^+^ exchanger	see Vagos et al. (2020)	2.5
3	Down-regulation of INaK	I*max*NaK	Maximum flux of the Na^+^-K^+^ pump	see Vagos et al. (2020)	0.5
4	Decreased Serca2a density	J*max*up	Maximum pump flux	0.0053	0.7
5	Increased RyR open probability	RyR_P[0]	RyR property to balance open probability and SR Ca^2+^ leak	0.2	2
		RyR_P[1]	RyR property to balance open probability and CaT amplitude	0.22	2
		RyR_P[5]	RyR single-channel open probability	8E-5	2
6	Decreased RyR density	RyR_P[11]	RyR2 density in SR fractions	0.007	0.25
		NRyRs	Number of ryanodine receptors per cluster	198000	0.25
7	Increased calcium buffering strength	Buff_factor	Calcium buffering factor	1.0	0.15
8	Full remodeling	All	–	–	–

### 2.3. Visualization and Analysis of Calcium Waves

Calcium wave propagation in the models was visualized in spatio-temporal plots of CaTs from each cell domain over time, similar to line scan images of individual myocytes. We also characterized individual remodeling effects by studying the occurrence of CaT amplitude alternans and calcium silencing. Calcium alternans arise from instabilities in the calcium handling system (Weiss et al., 2006; Gaeta and Christini, 2012; Xie et al., 2014), and have also been clinically associated with atrial arrhythmia (Franz et al., 2012). We defined alternans as beat-to-beat differences in CaT amplitude larger than 5%, while calcium silencing was defined as a ratio of central to membrane CaT amplitude (CaTc amplitude/CaTm amplitude) ≤ 0.10 for all beats. Based on the presence of alternans and calcium silencing, the individual models were classified into five different categories:

1. normal propagation;
2. calcium alternans;
3. calcium silencing;
4. alternans and calcium silencing;
5. unphysiological wave patterns (“other”).

Finally, we also report APD90 and resting memembrane potential (RMP) for the remodeled populations, since APD90 shortening and RMP depolarization have been linked to arrhythmogenic activity in cardiomyocytes (Bosch et al., 1999; Workman et al., 2001) and are therefore important quantities to characterize changes in phenotype due to remodeling.

## 3. Results

**Figure 3:**
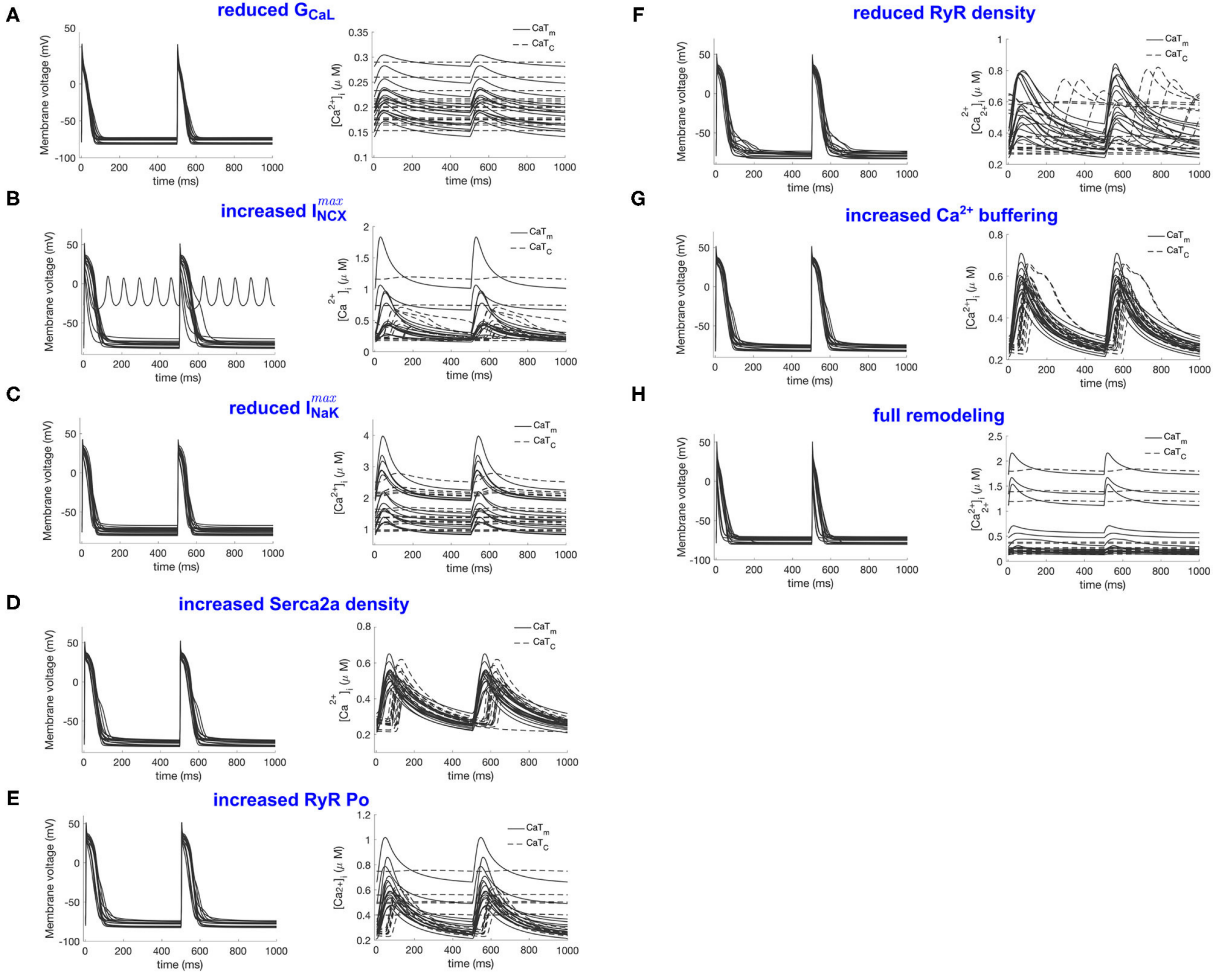
Illustration of the RAP remodeled populations. **(A–H)** Refer to the remodeled populations described in section 2.2, as indicated by the panel titles. For each population the action potentials are shown in the left panel and the CaTm and CaTc traces in the right panel.

In this section we list the effects of the remodeling alterations defined in Table 2, including both the individual mechanisms and the combined effect of all mechanisms. The results are summarized in Figure 3 and Table 3. Figure 3 includes APs and CaTs for the eight model populations defined in section 2.2. The individual changes observed in each population are described in more detail below.

**Table 2:** AP and CaT properties (mean ± std) of the control and remodeled populations.

**Population**	**APD90 (ms)**	**RMP (mV)**	**CaTA_**m**_(μM)**	**CaTA_**c**_(μM)**	**CaT_**ratio**_**	**CD50m(ms)**	**CD50c(ms)**
Control	96 ± 13	−78 ± 2	0.33 ± 0.07	0.32 ± 0.06	0.99 ± 0.20	94 ± 15	108 ± 28
GCaL(1)	65 ± 9	−76 ± 3	0.033 ± 0.006	0.0001 ± 0.00	0.0040 ± 0.0009	114 ± 10	71 ± 33
I*max*NCX(2)	113 ± 90	−73 ± 17	0.32 ± 0.23	0.15 ± 0.13	0.47 ± 0.38	77 ± 26	107 ± 61
I*max*NaK(3)	69 ± 10	−74 ± 4	0.76 ± 0.40	0.078 ± 0.07	0.086 ± 0.04	64 ± 8	159 ± 19
Serca2a dens. (4)	92 ± 13	−78 ± 3	0.28 ± 0.05	0.23 ± 0.06	0.84 ± 0.28	100 ± 17	99 ± 17
RyR Po (5)	91 ± 17	−77 ± 3	0.32 ± 0.08	0.17 ± 0.14	0.57 ± 0.45	90 ± 14	119 ± 36
RyR dens. (6)	107 ± 29	−78 ± 3	0.26 ± 0.13	0.064 ± 0.07	0.18 ± 0.20	119 ± 33	76 ± 56
calcium buff. (7)	93 ± 12	−78 ± 3	0.31 ± 0.06	0.28 ± 0.05	0.92 ± 0.19	96 ± 15	110 ± 20
full remodeling (8)	57 ± 19	−75 ± 3	0.13 ± 0.14	0.0061 ± 0.013	0.019 ± 0.030	102 ± 29	86 ± 77

**Table 3:** Effects of individually changing the RAP parameters in each of the 16 models of the control population.

**Population**	**Normal**	**Alternans**	**Silencing**	**Alt+Silenc**	**Other**
Control	100	0	0	0	0
GCaL(1)	0	0	100	0	0
I*max*NCX(2)	37.5	31.25	18.75	6.25	6.25
I*max*NaK(3)	37.5	0	62.5	0	0
Serca2a density (4)	87.5	12.5	0	0	0
RyR Po (5)	62.5	0	37.5	0	0
RyR density (6)	0	0	31.25	0	68.75
Calcium buffering strength (7)	100	0	0	0	0
Full RAP remodeling (8)	0	0	100	0	0

*Each row corresponds to one of the eight RAP populations, and the values correspond to % incidence of each type of calcium wave patterns in the population*.

**Figure 4:**
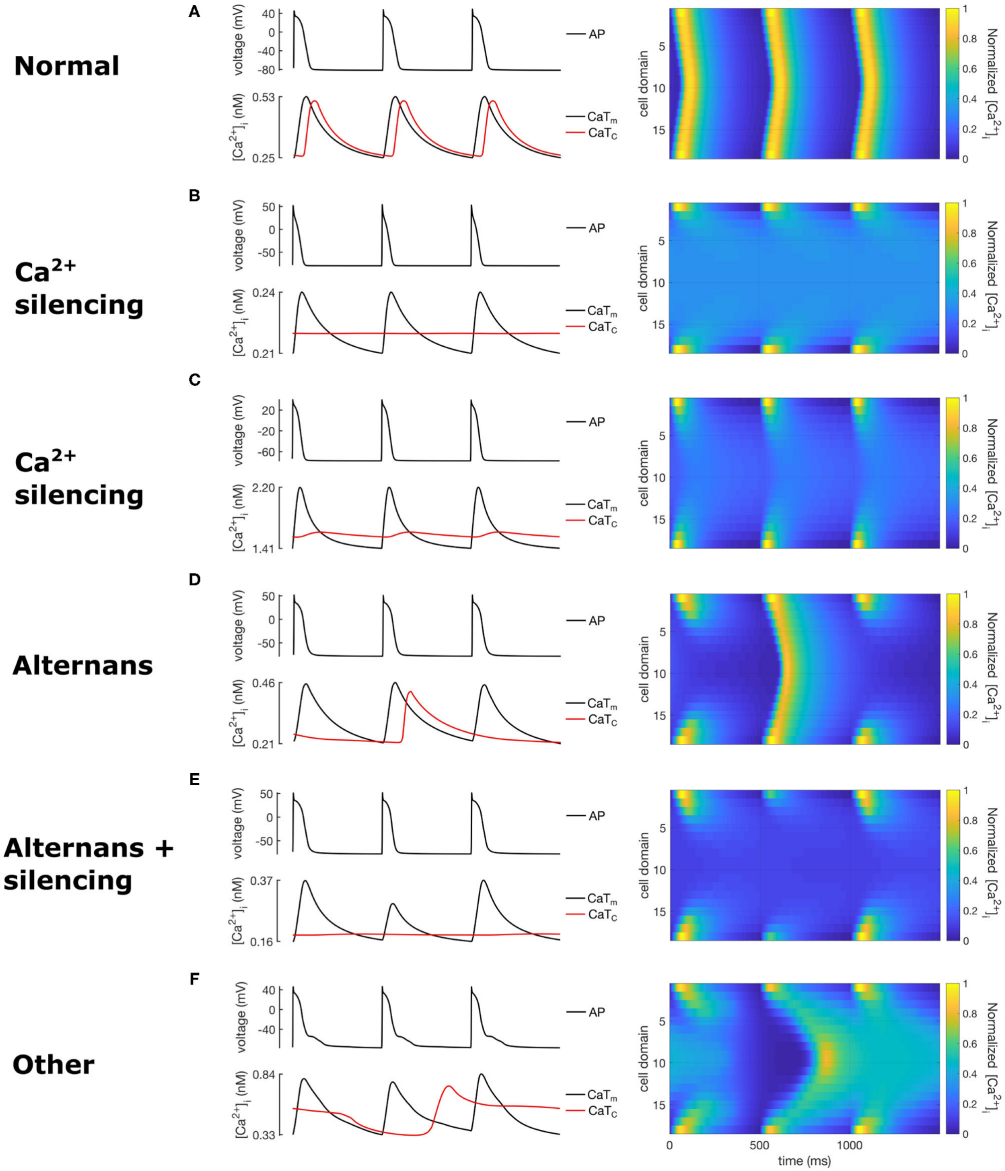
Exemplary calcium waves from the RAP remodeled populations showing calcium propagation patterns from the 5 wave categories. The left column shows AP and calcium transients, while the right column shows the corresponding spatio-temporal calcium wave plots. **(A)** corresponds to category 1 (normal), **(B,C)** to category 3, **(D)** to category 2, **(E)** to category 4, and **(F)** to category 5.

Table 3 classifies each population according to the characteristics described in section 2.3, while Figure 4 shows spatio-temporal plots of selected models to illustrate the characteristics of each category. For each row in Figure 4, the left panel shows APs and CaTs from the subsarcolemmal (CaTm) and central domain (CaTc), while the right panel shows spatio-temporal linescan plots illustrating the calcium propagation. Figure 4A show normal calcium propagation (category 1), Figures 4B,C show examples of calcium silencing (category 3), Figure 4D shows alternans (category 4), Figure 4E displays calcium silencing and alternans (cat 5), and Figure 4F shows a pattern we have characterized as unphysiological (category 5, “other”). The example in Figure 4A is from the control population, Figure 4B is from population 1 (reduced GCaL), Figure 4C from population 3 (reduced I*max*NaK), Figure 4D from population 4 (reduced Serca2a density), Figure 4E from population 2 (increased I*max*NCX), and Figure 4F is from population 5 (increased RyR open probability). The number of models falling into each category is quantified in Table 3. The table shows the percentage of models from each of the eight populations that fall into each category of calcium propagation.

### 3.1. Down-Regulation of I_**CaL**_

Figure 3A shows the population after reduction of GCaL to 40% in the population, which resulted in a consistent response of calcium silencing across all models. An example of a calcium wave from this population is shown in Figure 4B. Additionally, CaTm showed a considerably reduced amplitude compared to the control population, as would be expected since the amplitude of the sarcolemmal CaT is directly linked to the magnitude of ICaL. We also observed a significantly shortening of APD90 (see Table 2), which is a well-documented effect of reduced ICaL in myocytes.

### 3.2. Up-Regulation of I_**NCX**_

The effects of increasing I*max*NCX in the population resulted in a variety of calcium propagation patterns, including calcium alternans, silencing, and alternans and silencing (see Figure 3B). Additionally, this population showed large variations in APD90, with a mean APD90 larger than in the control population, as well as large variations in CaT amplitudes and diastolic calcium levels. Furthermore, one of the models failed to repolarize, as can be seen from the ripples in Figure 3B, left panel, with corresponding rippled CaTs (not shown). This model corresponded to a [Na+]i level of 41 mM, and [Ca2+]i of 5.8 μM. The perturbations in AP and CaTs observed in this population are not surprising given the known role of INCX in regulating both [Ca2+]i and repolarization (Weber et al., 2001).

### 3.3. Down-Regulation of INaK

Decreasing I*max*NaK resulted in about 60% of the models developing calcium silencing, while the remainder did not change significantly. This population showed significantly elevated [Na+]i levels (22 ± 4 mM vs. 9.5±2 mM, (two-sample *t*-test, α = 0.05), as expected given the decreased outflow of Na^+^ from the cell, which shifted the activity of INCX. As a consequence of reducing I*max*NaK, the forward mode (I_NCX, fwd_) and reverse mode (I_NCX, rev_) components of INCX were significantly smaller and larger, respectively, in the I*max*NaK population as compared to the control (two-sample *t*-test, α = 0.05). This resulted in an accumulation of calcium in the cytosol and in the SR. The RMP was also significantly more depolarized in this population.

The CaTm amplitude and diastolic calcium levels also varied considerably in this population (see Figure 3C and Table 2). There were no observable differences in the maximum conductances of the models that showed normal propagation versus calcium silencing, but found that models showing calcium silencing mostly corresponded to higher values of both [Na^+^]_i_ and [Ca^2+^]_SR_.

### 3.4. Decreased Serca2a Density

Decreasing Serca2a density to 70% of baseline value resulted in largely unchanged APs, and an overall small effect on CaTm and CaTc, with only about 20% of the models showing alternans and the others being mostly unaltered.

### 3.5. Increased RyR Open Probability

Increasing RyR open probability resulted in about 40% of the models developing calcium silencing. We did not observed any apparent associations between the occurrence of alternans in this population, and the values of the altered parameters, or of [Na+]i and [Ca2+]SR.

### 3.6. Decreased RyR Density

The effect of decreasing RyR density largely resulted in a variety of unphysiological calcium propagation patterns. One such wave pattern is shown in Figure 4F. This result indicates that the calcium system in this model is quite sensitive to such large changes of the two target parameters, NRyRs and RyR_P[11].

### 3.7. Increased calcium Buffering Strength

Increasing the calcium buffering strength in the population did not result in any form of abnormal calcium wave propagation, with all models showing a normal calcium propagation pattern. This is in contrast with experimental observations from Greiser et al. where the authors reported that an increased calcium buffering strength in the cell was likely to be correlated with the observed calcium silencing in the remodeled cells.

### 3.8. Full RAP Remodeling

Finally, we simultaneously incorporated all seven RAP remodeling mechanisms considered above. This change, incorporating all 10 parameter changes listed in Table 1, resulted in a population with significantly shorter APD90, reduced CaTm amplitude, and calcium silencing. This result is in good agreement with experimental observations in Greiser et al. (2014), where the authors observed calcium silencing in line scans of RAP remodeled cells, without the occurrence of alternans or other pathological calcium propagation behaviors.

## 4. Discussion

We used a previously developed population of 16 models representing normal rabbit atrial electrophysiology, and incorporated known remodeling effects from rapid atrial pacing. Seven new populations were built by changing parameters to reflect isolated physiological alterations, such as altered expression levels of an individual ionic current. Finally, one population incorporated the changes in all parameters simultaneously to reproduced full RAP-induced remodeling, as has been observed experimentally (Greiser et al., 2014).

The approach used here to quantify the effects of changing model parameters on model behavior differs from the more traditional approach where a single model is used to represent a certain region of the heart, animal species, or genotype. In contrast, we opted to use a small population of 16 models, obtained by calibration of a population of 3,000 models to experimentally measured rabbit electrophysiological parameters. An advantage of the population approach over a single model approach is that it allows to incorporate uncertainty and natural variability observed in the atrial myocytes, and may provide a more robust analysis of the effects of electrophysiological remodeling.

In our simulations each remodeled population showed different phenotypes, with some parameter changes leading to consistent changes across the entire population, while others resulting in a myriad of different phenotypes. The overall effects of individual parameter changes on calcium wave patterns are compiled in Table 3, while Table 2 summarizes the effects on APD90, RMP, and CaT in all model populations. Mean CaTm amplitude was significantly reduced in the populations with altered GCaL, I*max*NCX, I*max*NaK, and RyR_P[11], while CaTc amplitude was reduced in all populations, except the one with altered calcium buffering. These amplitude changes resulted in an overall reduced CaTratio in the populations with altered GCaL, I*max*NCX, I*max*NaK, Serca2a density, RyR Po, and RyR density.

In our simulations calcium silencing was mainly driven by reduction of GCaL and I*max*NaK, when these parameters are changed individually. The similarities between the GCaL and full remodeling populations indicate that the 60% reduction of GCaL was the dominant effector of changes in AP and CaT morphologies. Furthermore, despite significant differences in APD90 and RMP among the various remodeled populations, with one model showing an afterdepolarization and another showing failed repolarization, APs were mostly consistent across the populations. Greater differences were observed in CaTm and CaTc morphologies, especially in the GCaL, I*max*NCX, RyR Po, RyR density, and full remodeling populations. Furthermore, modification of GCaL, Serca2a density, Buff_factor, and full remodeling resulted in consistent alterations in calcium wave propagation patterns, while changes in the other parameters entailed a more complex response, despite considerable differences in the values of their maximum ionic conductances. In contrast, changes in the other parameters which resulted in a more varied set of responses indicate that those parameters play a more complex role in the calcium handling system, where the values of maximum ionic conductances may also have had an impact.

Experimental observations from Greiser et al. (2014) suggested that the main driver of calcium silencing in rabbit atrial cells could be increased calcium buffering strength, whereas the reduction in ICaL was unlikely to play a significant role. Our computer simulations of the full remodeling case, which aims to emulate the array of cellular alterations measured experimentally, are consistent with the experimental observations of calcium silencing following RAP remodeling. However, our results indicate that reduced ICaL, rather than increased calcium buffering strength, may be the main effector of calcium silencing. calcium alternans were only observed in two of the remodeled populations (I*max*NCX and Serca2a density), and in a fairly low percentage of the models. This low occurrence of alternans suggests that they are the result of more drastic changes in cellular mechanisms, which were not captured in the remodeled populations constructed here. Overall, the results presented here provide insight into the possible mechanisms of calcium silencing in rabbit atrial myocytes.

As with any modeling study, the approach developed here has a number of limitations and assumptions. We note that, although the approach proposed here, whereby variability in maximum ionic conductances is taken into account, can improve robustness and reliability of simulation results, the control population used in this study is only an abstraction for a real population of myocytes obtained from different specimens and varying regions of the heart. The control population was produced by experimentally calibrating a population of 3,000 models against rabbit AP and CaT characteristics (Vagos et al., 2020), and thus the variation in maximum conductances introduced in the population cannot be verified experimentally as of this point due to the lack of data. A more holistic approach would be to take into account experimentally observed variability in ionic channel expression levels, for instance, and to calibrate the simulated population accordingly. The populations could also be expanded via re-sampling of the maximum conductances, such as introducing perturbations around the baseline values, and recalibrating.

While the RAP populations were based on previously published changes in ionic currents and calcium dynamics during RAP and AF, there is considerable variability in the reported changes, see for instance (Greiser et al., 2011). Our modifications are mainly based on experimental data from Greiser et al. (2014), and while all the modifications are supported by experimental data they are by no means the only possible alternative. A natural extension of the present work would be to extend the population of models approach to consider a wider range of previously reported electrophysiological remodeling, to capture more of the underlying uncertainty and potentially gain insight into plausible mechanisms underlying the observations. Another very natural extension would be to incorporate variability in the RyR parameters as well as ${\text{J}}_{\text{up}}^{max}$ and Buff_factor, thereby producing a population that more reliably captures the full spectrum of calcium wave propagation patterns resulting from RAP remodeling. Such an extension could also be complemented by a more detailed quantification and characterization of the calcium propagation properties, providing more detailed insight than the broad categorization performed here.

## 5. Conclusions

In this study we have proposed an approach for analyzing the effects of rapid atrial pacing remodeling in a population of healthy rabbit atrial myocytes. The results indicate that reduced ICaL is the main determinant of the occurrence of calcium silencing, although decreased I*max*NCX, increased I*max*NaK, increased RyR open probability, and reduced RyR density also resulting in some cases of calcium silencing. Reduction in INCX, and in Serca2a density gave rise to calcium alternans in a small percentage of instances, while changes in RyR density largely resulted in unphysiological calcium transients. The methodology proposed in this paper provides a robust framework for assessing effects of cellular remodeling under conditions of uncertainty and natural variability, and offers additional insight compared with studies based on a single representative model.

## Data Availability Statement

Parameter sets and simulation data presented in this study can be found at: https://github.com/marciavagos/rabbit_model_RAP_data.git.

## Author Contributions

MV contributed to conceiving the study, implemented the model, performed all numerical simulations and data analyses, wrote the first draft of all sections and revised, and edited all sections. JS and HA contributed to conceiving the study, contributed to the analysis and model development, and revised and edited all sections of the paper. US and JH contributed to conceiving the study and revised and edited all sections of the paper. All authors contributed to the article and approved the submitted version.

## Conflict of Interest

The authors declare that the research was conducted in the absence of any commercial or financial relationships that could be construed as a potential conflict of interest.

## Footnotes

^1^See https://github.com/marciavagos/Rabbit_model.git

## References

[B1] Aslanidi O. Boyett M. Dobrzynski H. Li J. Zhang H. (2009). Mechanisms of transition from normal to reentrant electrical activity in a model of rabbit atrial tissue: interaction of tissue heterogeneity and anisotropy. Biophys. J. 96, 798–817. 10.1016/j.bpj.2008.09.057 19186122 PMC3325123

[B2] Bootman M. D. Smyrnias I. Rdiger Coombes S. Roderick H. L. (2011). Atrial cardiomyocyte calcium signalling. Biochim. Biophys. Acta 1813, 922–934. 10.1016/j.bbamcr.2011.01.030 21295621

[B3] Bosch R. F. Zeng X. Grammer J. B. Popovic K. Mewis C. Kuhlkamp V. (1999). Ionic mechanisms of electrical remodeling in human atrial fibrillation. Cardiovasc. Res. 44, 121–131. 10.1016/S0008-6363(99)00178-9 10615396

[B4] Franz M. R. Jamal S. M. Narayan S. M. (2012). The role of action potential alternans in the initiation of atrial fibrillation in humans: a review and future directions. Europace 14(Suppl. 5):v58-v64. 10.1093/europace/eus273 23104916 PMC3697801

[B5] Gaeta S. A. Christini D. J. (2012). Non-linear dynamics of cardiac alternans: subcellular to tissue-level mechanisms of arrhythmia. Front. Physiol. 3:157. 10.3389/fphys.2012.00157 22783195 PMC3389489

[B6] Grandi E. Maleckar M. M. (2016). Anti-arrhythmic strategies for atrial fibrillation: the role of computational modeling in discovery, development, and optimization. Pharmacol. Ther. 168, 126–142. 10.1016/j.pharmthera.2016.09.012 27612549 PMC5140742

[B7] Greiser M. (2017). Calcium signalling silencing in atrial fibrillation. J. Physiol. 595, 4009–4017. 10.1113/JP273045 28332202 PMC5471362

[B8] Greiser M. Kerfant B.-G. Williams G. S. Voigt N. Harks E. Dibb K. M.. (2014). Tachycardia-induced silencing of subcellular ca2+ signaling in atrial myocytes. J. Clin. Invest. 124, 4759–4772. 10.1172/JCI70102 25329692 PMC4347234

[B9] Greiser M. Lederer W. J. Schotten U. (2011). Alterations of atrial Ca2+ handling as cause and consequence of atrial fibrillation. Cardiovasc. Res. 89, 722–733. 10.1093/cvr/cvq389 21159669

[B10] Narayan S. M. Bode F. Karasik P. L. Franz M. R. (2002). Alternans of atrial action potentials during atrial flutter as a precursor to atrial fibrillation. Circulation 106, 1968–1973. 10.1161/01.CIR.0000037062.35762.B4 12370221

[B11] Smith G. L. Eisner D. A. (2019). Calcium buffering in the heart in health and disease. Circulation 139, 2358–2371. 10.1161/CIRCULATIONAHA.118.039329 31082292 PMC6520234

[B12] Trafford A. W. Clarke J. D. Richards M. A. Eisner D. A. Dibb K. M. (2013). Calcium signalling microdomains and the t-tubular system in atrial mycoytes: potential roles in cardiac disease and arrhythmias. Cardiovasc. Res. 98, 192–203. 10.1093/cvr/cvt018 23386275

[B13] Trayanova N. A. (2014). Mathematical approaches to understanding and imaging atrial fibrillation: significance for mechanisms and management. Circ. Res. 114, 1516–1531. 10.1161/CIRCRESAHA.114.302240 24763468 PMC4043630

[B14] Vagos M. van Herck I. G. M. Sundnes J. Arevalo H. J. Edwards A. G. Koivumäki J. T. (2018). Computational modeling of electrophysiology and pharmacotherapy of atrial fibrillation: recent advances and future challenges. Front. Physiol. 9:1221. 10.3389/fphys.2018.01221 30233399 PMC6131668

[B15] Vagos M. R. Arevalo H. Heijman J. Schotten U. Sundnes J. (2020). A novel computational model of the rabbit atrial cardiomyocyte with spatial calcium dynamics. Front. Physiol. 11:1205. 10.3389/fphys.2020.556156 33162894 PMC7583320

[B16] Voigt N. Heijman J. Qiongling Chiang D. Y. Li N. Karck M.. (2014). Cellular and molecular mechanisms of atrial arrhythmogenesis in patients with paroxysmal atrial fibrillation. Circulation 129, 145–156. 10.1161/CIRCULATIONAHA.113.006641 24249718 PMC4342412

[B17] Weber C. R. Ginsburg K. S. Philipson K. D. Shannon T. R. Bers D. M. (2001). Allosteric regulation of na/ca exchange current by cytosolic ca in intact cardiac myocytes. J. Gen. Physiol. 117, 119–131. 10.1085/jgp.117.2.119 11158165 PMC2217247

[B18] Weiss J. N. Karma A. Shiferaw Y. Chen P.-S. Garfinkel A. Qu Z. (2006). From pulsus to pulseless. Circ. Res. 98, 1244–1253. 10.1161/01.RES.0000224540.97431.f0 16728670

[B19] Workman A. J. Kane K. A. Rankin A. C. (2001). The contribution of ionic currents to changes in refractoriness of human atrial myocytes associated with chronic atrial fibrillation. Cardiovasc. Res. 52, 226–235. 10.1016/S0008-6363(01)00380-7 11684070

[B20] Xie Y. Izu L. T. Bers D. M. Sato D. (2014). Arrhythmogenic transient dynamics in cardiac myocytes. Biophys. J. 106, 1391–1397. 10.1016/j.bpj.2013.12.050 24655514 PMC3984988

